# Editorial for the Micro/Nanoscale Electrokinetics Section

**DOI:** 10.3390/mi15121414

**Published:** 2024-11-25

**Authors:** Xiangchun Xuan

**Affiliations:** Department of Mechanical Engineering, Clemson University, Clemson, SC 29634, USA; xcxuan@clemson.edu

Electrokinetics is the study of fluid flow and particle motion driven by electricity [1]. The most prominent electrokinetic phenomena are electroosmosis, electrophoresis, streaming potential and sedimentation potential, where a liquid moves tangentially to a charged solid surface in the presence of an electric field [2]. All these phenomena are associated with the electric double layer (EDL) that is spontaneously formed near a charged solid surface [3]. Electrokinetics is often the method of choice in micro/nanofluidic devices because of its favored downscaling as well as the ease, precision, and autonomy of operation compared to the traditional hydrodynamic method [4]. It has been extensively used to transport and manipulate fluids and various types of particles to achieve lab-on-a-chip applications in many areas such as biomedical, chemical, environmental and forensic etc. [5,6]. 

In recognizing the significance and relevance of electrokinetics, I am pleased to announce a new section, Micro/Nanoscale Electrokinetics, in the journal of *Micromachines*. This section welcomes original research and review articles on either a fundamental investigation or an applicational exploration of any electrokinetic phenomena in micro/nanofluidic devices. The electrokinetic phenomena of interest include, but are not necessarily limited to, electroosmosis, electrophoresis, induced-charge electrokinetics, dielectrophoresis, electro-rotation, electro-hydrodynamics, electrothermal flow, diffusioosmosis, diffusiophoresis, streaming potential/current, and electrokinetic energy conversion etc. in both Newtonian and non-Newtonian fluids.

In this Editorial, I aim to highlight a few trending research directions in the field of micro/nanoscale electrokinetics, which hopefully will pave the way to stimulate further studies in these directions and as well more new directions.

## 1. Nonlinear Electroosmosis and Electrophoresis in Newtonian Fluids

In classical electrokinetics with Newtonian fluids, electroosmosis and electrophoresis are both proportional and parallel to the imposed electric field regardless of DC or AC [7]. These linear dependences, however, break down around curved solid surfaces because of surface conduction in the EDL [8,9]. Concentration–polarization electroosmosis [10] is induced around stationary dielectric surfaces under low-frequency AC electric fields. This nonlinear electroosmosis also occurs around dielectric particles and has been utilized for particle focusing [11] and separation [12]. Nonlinear electrophoresis has been predicted and observed for highly charged dielectric particles under large DC electric fields because of the non-uniformity of surface conduction over curved particle surfaces [13,14]. Its velocity depends on the particle size, charge, and shape [15,16,17,18], which plays a significant role in characterizing and separating particles in electrokinetic systems [19,20,21,22].

## 2. Nonlinear Electroosmosis and Electrophoresis in Non-Newtonian Fluids

As many of the chemical and biological samples in micro/nanofluidic applications are complex fluids exhibiting non-Newtonian characteristics [23], there have been extensive theoretical and/or numerical studies on electroosmosis and electrophoresis in non-Newtonian fluids over the past two decades [24,25,26]. Both nonlinear electroosmosis and nonlinear electrophoresis have been predicted to occur because of fluid shear thinning and/or elasticity effects [24,27,28]. A recent experiment in shear thinning xanthan gum solutions [29] confirmed these nonlinear electric field dependences, where the nonlinear index values of electroosmotic, electrophoretic, and electrokinetic velocities all increase with increasing polymer or buffer concentration. In another experimental study with viscoelastic poly(ethylene oxide) solutions [30], the fluid elasticity effect was demonstrated to induce the particle size dependence of electrophoretic velocity. 

## 3. Dielectrophoresis-Enabled Single Cell Analysis

Dielectrophoresis (DEP) has been extensively used to manipulate particles ranging from microscopic cells through mesoscopic viruses to nanoscopic molecules [31,32]. It has the capability to capture and/or release individual objects for single cell analysis. Positive DEP has been demonstrated to guide and position single cells within lithographically defined wells on both glassy carbon and gold microelectrodes [33]. It take a much longer time to attract suspended cells into the wells than precipitated cells because of the quickly decayed DEP force away from the electrode. This issue can be resolved by using 3D electrodes that are the same height as the microchannel [34]. Droplets have also been demonstrated for potential single cell analysis, where cell-encapsulated droplets can be hydrodynamically trapped into different pockets and then selectively extracted from the pockets by the DEP force [35].

## 4. Protein Dielectrophoresis

Protein DEP has the potential to make a strong impact both as an enrichment method in bioanalysis and as a manipulation tool for bionanotechnological applications [36]. The responses of protein DEP can be quantified through either fluorescent probes or electrical measurements such as impedance spectroscopy [37]. However, the reported DEP data for over twenty different globular proteins cannot be explained using the standard DEP theory, where the calculated DEP force is too small to overcome the Brown motion of proteins [38]. A molecular dynamics simulation-supported empirical theory has been proposed to consider both the induced and permanent dipole moments of proteins, yielding a molecular version of the traditional Clausius–Mossotti function that is derived from macroscopic electrostatics [39,40]. Another theoretical attempt in this direction is looking at DEP from the system’s point of view [41,42,43]. 
